# Impacts of Cross-Regional Transport on Ozone Pollution in the Fen-Wei Plain: Insights from Multi-Source Observations and Model Simulation

**DOI:** 10.3390/toxics14030189

**Published:** 2026-02-24

**Authors:** Yufei Han, Danni Xu, Anjie Yin, Chang Liu, Yuheng Chen, Kaihui Zhao

**Affiliations:** 1 Yunnan Key Laboratory of Meteorological Disasters and Climate Resources in the Greater Mekong Subregion, Yunnan University, Kunming 650091, China; 12025118065@stu.ynu.edu.cn (Y.H.); zz2331@ynufe.edu.cn (D.X.); 12024118067@stu.ynu.edu.cn (A.Y.); chenyuheng@stu.ynu.edu.cn (Y.C.); 2 Information School, Yunnan University of Finance and Economics, Kunming 650221, China

**Keywords:** PM_2.5_ and O_3_, source apportionment, Fenwei Plain, multi-source 3D observations, regional transport

## Abstract

Unfavorable terrain conditions and intensive emissions have led to a deteriorating trend of ozone (O_3_) pollution in the Fenwei Plain (FWP), which has attracted increasing attention. However, the lack of observations and Volatile Organic Compound (VOC) component observation data has seriously constrained an in-depth understanding of the formation mechanisms of O_3_ pollution. The multi-source observations conducted in this study provides first-hand evidence for characterizing the evolution of O_3_ pollution in the FWP. O_3_ lidar vertical profiles reported high-concentration layers exceeding 130 µg/m^3^, O_3_ vertical flux high-concentration layers exceeding −50 µg/(m^2^⋅s), confirming downward transport to the surface. The VOCs components were dominated by Oxygenated Volatile Organic Compounds (OVOCs) (>300 ppbv) and alkanes (>20 ppbv). O_3_ source apportionment technology analysis indicated transport from the Henan (HN) and Hubei (HB) contributed 24.99% and 40.02% of surface O_3_ enhancement. Interestingly, a close linkage between O_3_ precursor sensitivity (OPS) variations and contribution from potential source regions was noticed. Large contributions from HN and HB drove the OPS toward a VOC-limited regime, with a concurrent drop in the HCHO/NO_2_ indicator to 1.73. Our results underscore the great importance of the impacts of regional transport on OPS from different source areas when formulating strategies for regional joint prevention and control.

## 1. Introduction

Tropospheric O_3_, a typical secondary air pollutant, exerts detrimental effects on human health, terrestrial ecosystems, and global climate change [1,2,3]. Furthermore, due to its strong oxidative potential properties, it can adversely affect vegetation by reducing stomatal conductance and carbon assimilation rates, consequently compromising the carbon sequestration capacity of terrestrial ecosystems [4]. Over the past decade, China has achieved remarkable success in mitigating particulate matter with <2.5 µm aerodynamic diameters (PM_2.5_) pollution. In contrast, O_3_ pollution has increasingly emerged as a severe and complex environmental challenge. Particularly in densely populated megacity clusters, O_3_ concentrations have shown a rising trend that runs counter to the decline in PM_2.5_ [5,6]. The combined pollution characteristics of O_3_ and PM_2.5_ further exacerbate the complexity of air quality management, highlighting the urgent need for more integrated and synergistic control strategies [7].

As a complex atmospheric challenge, O_3_ pollution is intensifying globally due to multiple interconnected drivers. Extreme wildfires are a direct source of severe ozone episodes [8,9]. Furthermore, climate anomalies such as droughts can counteract pollution control measures, leading to rising urban O_3_ despite lower emissions [10]. Simultaneously, transboundary transport accounts for a substantial fraction of health impacts, illustrating the need for international cooperation [11]. Future climate warming is projected to worsen this pollution, continuously endangering health and agriculture [12]. As a newly emerging hotspot for O_3_ pollution in China, the FWP is characterized by unfavorable topographic conditions and intensive emissions from the energy, chemical, and transportation sectors. This combination results in a severely constrained atmospheric capacity [13,14] and particularly prominent pollution issues [15,16]. O_3_ pollution in this region exhibits not only high concentrations and long duration but is also significantly modulated by altitude and local topography [16,17]. The formation mechanism is generally attributed to the complex interplay between local photochemical production and regional transport processes [18]. Therefore, a thorough analysis of the formation mechanisms of severe O_3_ episodes in the FWP is crucial for developing effective regional joint prevention and control strategies.

It is widely acknowledged that the combination of adverse meteorological conditions and regional transport plays a key role in triggering severe O_3_ pollution episodes [18]. In the topographically constrained FWP, high temperatures, intense solar radiation, and stagnant synoptic conditions not only accelerate local photochemical processes but also often coincide with specific large-scale circulation patterns, such as high-pressure back (HPB) and Northeast China low–Northwest Pacific high pattern. HPB and Northeast China low–Northwest Pacific high are identified as two dominant synoptic patterns leading to O_3_ pollution in the FWP. The HPB pattern, characterized by a surface high-pressure system, promotes O_3_ production through atmospheric subsidence and clear skies [19]. The Northeast China low—Northwest Pacific high is most favorable for O_3_ formation due to high temperature and low humidity conditions [20]. These patterns facilitate long-range transport, which significantly contributes to the initiation and peak concentrations of O_3_ pollution [21,22]. To unravel these complex physical and chemical interactions, current research largely depends on simulations conducted with chemical transport models (CTMs) such as WRF-Chem and CMAQ [23,24]. Such modeling efforts have effectively captured the spatial distribution of pollutants and provided preliminary quantifications of the impact of regional transport on downwind air quality, thereby laying the theoretical groundwork for regional coordinated control strategies.

Regarding source apportionment studies in Xianyang City (XY (108.7° E, 34.3° N)), existing research has primarily employed the HYSPLIT backward trajectory model [25,26,27]. While this method can effectively trace potential geographical source regions, it often fails to disentangle the specific sectoral emission characteristics, such as those from industry and transportation, within complex mixed air masses. As a result, the quantitative contribution of different emission sectors to downwind O_3_ formation during regional transport events remains unclear. However, a critical gap remains in understanding how regional transport simultaneously influences secondary aerosol formation and shifts in photochemical sensitivity in downwind areas. Addressing this requires the integration of multi-source observations to deliver direct evidence for elucidating O_3_ formation mechanisms.

To further investigate the aforementioned issues, this study focuses on a representative city of the FWP—Xianyang—and examines a typical severe O_3_ pollution episode that occurred in September 2024. By integrating multi-source observational data, including ground-based air quality and meteorological monitoring networks, O_3_ lidar and wind profile radar vertical sounding, and high spatiotemporal resolution geostationary satellite remote sensing (GEMS), together with the HYSPLIT model and the NAQPMS model, this research aims to achieve the following objectives: (1) directly and quantitatively assess the horizontal and vertical O_3_ transport fluxes during the pollution episode based on 3D observations; (2) elucidate the impacts of regional transport on atmospheric oxidative capacity (AOC), secondary component formation, and O_3_ formation sensitivity [28,29]; (3) achieve a dual quantitative attribution of pollution sources across geographic regions and emission sectors. The findings will provide robust scientific support for a deeper understanding of transport-driven O_3_ pollution mechanisms and for the formulation of targeted, effective regional joint prevention and control strategies.

In this study, a set of three-dimensional observational data combined with an advanced air quality model was employed to elucidate the formation mechanisms of O_3_ pollution episodes in the FWP. A representative O_3_ pollution event was analyzed to quantify the relative contributions of regional transport and local photochemical production. The specific objectives are to (1) quantify the contributions of different sectors and geographic regions to surface O_3_ enhancement; (2) conduct a comprehensive assessment of the key drivers governing O_3_ episodes over the FWP; and (3) determine the temporal variations in AOC and O_3_ precursor sensitivity (OPS) [30].

## 2. Materials and Methods

### 2.1. Data Sources

#### 2.1.1. Observational Data

Hourly surface observation data, including 115 VOC species, PM_2.5_ components, O_3_, NO_2_, SO_2_, temperature, relative humidity (RH), wind direction (WD), and wind speed (WS), as well as vertical profiles from O_3_ lidar and wind profile radar, were obtained from the Shaanxi Environmental Monitoring Center Station (Xi’an, China, https://sthjt.shaanxi.gov.cn/, accessed on 7 September 2025).

VOC measurements were performed using gas chromatography–mass spectrometry (GC–MS), while water-soluble ions in PM_2.5_ were analyzed by ion chromatography. Various remote sensing instruments were used to characterize vertical distributions of wind speed and direction, and O_3_. These instruments include the model CASEO-O_3_-LIDAR O_3_ lidar and the model S6000 wind profiling lidar in XY.

Quality control procedures, implemented in collaboration with local monitoring stations, included the removal of outliers and interpolation of missing values using linear methods [31,32]. Following the Technical Regulation on Ambient Air Quality Index (on trial) (HJ 633-2012) [33] and the Ambient Air Quality Standards (GB 3095-2012) [34], an O_3_ pollution day was defined as having a daily maximum 8 h average (MDA8) O_3_ concentration exceeding 160 µg/m^3^.

#### 2.1.2. Reanalysis Data

The meteorological data for synoptic analysis over XY and its surrounding areas were obtained from the European Centre for Medium-Range Weather Forecasts (ECMWF, Reading, UK) Fifth-Generation Global Atmospheric Reanalysis (ERA5; available at https://cds.climate.copernicus.eu/datasets, accessed on 8 September 2025). The dataset contains sea-level pressure, 10 m horizontal winds, as well as geopotential height and horizontal wind speed at 850 hPa and 700 hPa, with a horizontal resolution of 0.25° × 0.25° and a vertical resolution of 37 pressure levels. For the HYSPLIT calculations, meteorological input fields were taken from the September 2024 archive of the NOAA Air Resources Laboratory (ARL, College Park, MD, USA; https://www.ready.noaa.gov/archives.php, accessed on 9 October 2025), which also features a horizontal resolution of 0.25° × 0.25° and a vertical resolution of 56 pressure levels.

#### 2.1.3. Satellite Data

NO_2_ and HCHO vertical column densities were obtained from the Geostationary Environment Monitoring Spectrometer (GEMS, Incheon, Republic of Korea), which is the first UV-Vis hyperspectral imaging spectrometer in geostationary orbit. Launched in February 2020 on the GK2B satellite and positioned at 128.2° E, GEMS provides continuous daytime hourly observations over East Asia with a spatial resolution of approximately 3.5 km × 8 km. In this study, we utilized the operational Level-2 (L2) version 2.0 products. The NO_2_ retrieval employs the Differential Optical Absorption Spectroscopy method within the 432–450 nm fitting window, while HCHO are derived from the 320–360 nm range. To ensure data quality, observations with a cloud fraction > 0.3 or a solar zenith angle > 70° were excluded from the analysis to minimize retrieval uncertainties. Due to data gaps on 24 September, GEMS observations at 04:45 UTC and 07:45 UTC were utilized for NO_2_ and HCHO, respectively. For the remainder of the study period, GEMS data at 06:45 UTC were consistently employed to ensure temporal representative.

### 2.2. Methods

#### 2.2.1. Hybrid Single-Particle Lagrangian Integrated Trajectory (HYSPLIT)

The HYSPLIT model was applied to elucidate the potential sources of atmospheric pollutants in XY. HYSPLIT, jointly developed by the National Oceanic and Atmospheric Administration (NOAA) and the Australian Bureau of Meteorology, is a widely utilized system in regional pollution research. In this study, the HYSPLIT model (v5.0.0, https://www.ready.noaa.gov/HYSPLIT_applehysp.php, accessed on 30 October 2025) was employed to obtain air mass trajectory data for XY during 21–30 September 2024, and MeteoInfo (v4.1.3) was subsequently used for creating trajectory maps. These air mass trajectories were utilized to validate the accuracy of the O_3_ source apportionment method (OSAM) results. XY (34.330° N, 108.700° E) was selected as the terminal point for model simulations or particle reception points. The initial altitude for backward trajectory calculation was set at 500 m. The simulation of the pollution period included the calculation of 48 h backward air mass trajectories arriving at the simulation endpoint at 00:00, 03:00, 06:00, 09:00, 12:00, 15:00, 18:00, and 21:00 each day. Several studies have demonstrated that HYSPLIT can accurately track the sources of O_3_ and PM_2.5_ in key cities of the FWP [35,36].

#### 2.2.2. Nested Air Quality Prediction Modeling System (NAQPMS)

The O_3_ source apportionment in Xianyang (XY) during September 2024 was conducted using the OSAM within the nested air quality prediction modeling system (NAQPMS). Based on an online tracer-tagging technique, OSAM tracks tagged emissions of O_3_ precursors from specified source regions and sectors throughout the simulation, while preserving mass conservation and without perturbing the base chemistry. By explicitly following atmospheric photochemical mechanisms, the module quantifies the transformation and transport of these tagged species, thereby attributing the resulting O_3_ formation to their respective sources. The model was configured with 46 vertical layers extending from the surface to 50 hPa, with the lowest layer height set at approximately 40 m. This approach effectively addresses nonlinearities in atmospheric chemistry and allows for a clear separation of local photochemical production from regional transport contributions [37,38].

## 3. Results and Discussion

### 3.1. General Characteristics of O_3_ Episode in September 2024

**Figure 1:**
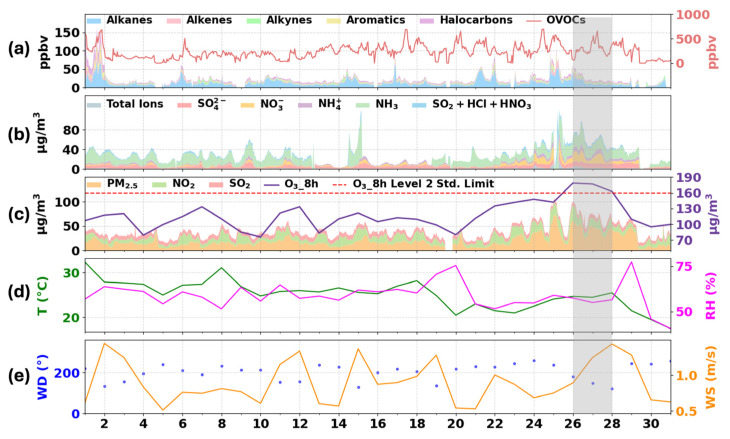
Time series of air pollutants, and meteorological parameters in XY during 1 September to 1 October 2024. (**a**) Concentrations of different VOC species (ppbv), with OVOCs plotted on the right axis; (**b**) mass concentrations (µg/m^3^) of major water-soluble ions and other components in PM_2.5_; (**c**) mass concentrations (µg/m^3^) of PM_2.5_, NO_2_, SO_2_, and MDA8 O_3_. The red dashed line denotes the national Grade II ambient air quality standard limit for O_3_ (160 µg/m^3^); (**d**) temperature (T) and relative humidity (RH); (**e**) surface wind speed (WS) and wind direction (WD). The gray shaded area highlights the severe O_3_ episode that occurred from 26 to 28 September.

Figure 1 presents the time series of major air pollutants, PM_2.5_ chemical components, and key meteorological parameters in XY from 1 September to 1 October 2024. Notably, a three-day O_3_ pollution episode persisted from 26 to 28 September (gray-shaded area). During this period, the average MDA8 O_3_ concentration reached 170 µg/m^3^, consistently exceeding the National Ambient Air Quality Grade II standard (160 µg/m^3^) and reaching 180% of the average level during clean periods (99 µg/m^3^ on 20–21 and 29–30 September). MDA8 O_3_ increased rapidly on 25 September, peaking at 173 µg/m^3^ on 26 September (Figure 1c).

During the O_3_ episode, PM_2.5_ concentrations rose markedly from an average of 10.21 µg/m^3^ during clean days to 34.42 µg/m^3^, indicating clear co-pollution behavior between PM_2.5_ and O_3_. Analysis of PM_2.5_ chemical composition (Figure 1b) reveals that this increase was driven mainly by secondary pollutants. For instance, NO_3_^−^ peaked at 26.95 µg/m^3^ on 25 September—about 270% higher than during clean periods (<1 µg/m^3^), and remained elevated at 12.45 µg/m^3^ throughout the episode. Similar upward trends were observed for SO_4_^2−^, NH_4_^+^, and gaseous acidic species (SO_2_ + HCl + HNO_3_). This intense secondary formation was closely linked to the accumulation of O_3_ precursors. As shown in Figure 1a, total volatile organic compounds (TVOCs) began rising synchronously with PM components from 22 September, increasing from ~80 ppbv in clean periods to nearly 300 ppbv on average, with a peak exceeding 700 ppbv on 25 September before declining in the afternoon of 26 September and remaining low afterward. In contrast, NO_2_ levels stayed relatively high (Figure 1c), providing a continual supply of precursors for secondary formation. Interestingly, from 22 to 25 September, while precursors were accumulating, MDA8 O_3_ remained below 160 µg/m^3^ and its rate of increase gradually slowed. This pattern likely reflects the stagnant meteorological conditions, characterized by low temperature, low humidity, and weak winds (Figure 1d,e), which suppressed both pollutant dispersion and photochemical activity during these days [39,40]. These conditions favored the early-stage buildup of local precursors, resulting in the observed gradual rise in O_3_ and its precursors before the full outbreak of the pollution episode.

As illustrated in Figure 1d, the meteorological conditions displayed patterns atypical of locally driven photochemical production during this pollution episode. Notably, the mean surface air temperature from 26 to 28 September (23.5 °C) was lower than that during the preceding clean period of 21–22 September (26.0 °C). This contrasts with the conventional understanding that O_3_ pollution is typically driven by high temperatures and strong solar radiation, which promotes local photochemistry [41]. The observed temperature anomaly suggests that the dominant mechanism behind this event was likely not local production, but rather the rapid influx of an externally transported polluted air mass [42,43].

**Figure 2:**
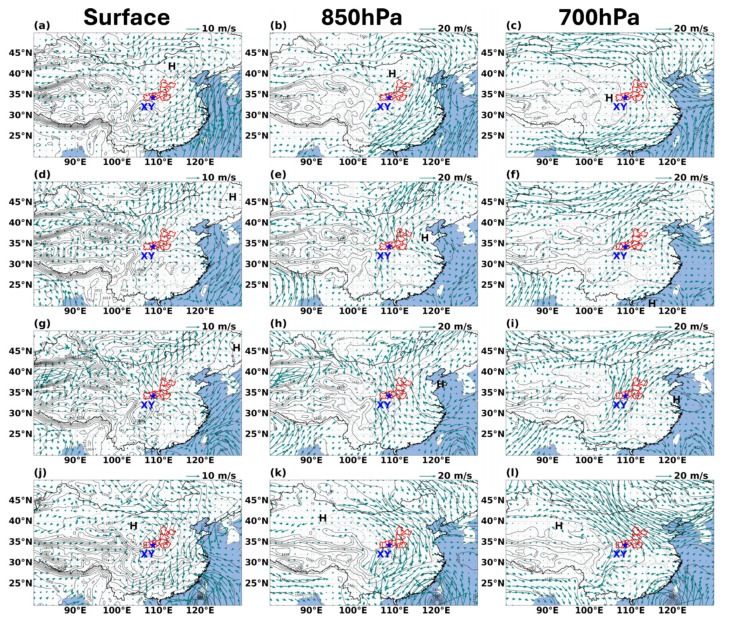
Synoptic circulation patterns of sea-level pressure, 10 m wind speed, and geopotential height at surface, 850 hPa and 700 hPa for typical clean days on 21 September (**a**–**c**) and 30 (**j**–**l**), as well as typical polluted days on September 26 (**d**–**f**) and 27 (**g**–**i**). The asterisk denotes the central location of XY. The red area is FWP. “H” indicates high pressure.

Numerous studies have highlighted the critical role of large-scale synoptic circulation and its induced dynamic conditions in driving regional O_3_ pollution [18]. To examine this influence, 26–27 September were selected as representative polluted days, 21 and 30 September as clean days, and 23–25 September as the pre-outbreak phase. The corresponding synoptic situations at the surface, 850 hPa, and 700 hPa are shown in Figure 2. These circulation patterns collectively illustrate how synoptic-scale dynamics fundamentally shaped the evolution of this pollution episode.

During 26–27 September, XY and its surrounding areas were under the influence of southeasterly airflow on the western flank of a continental high-pressure system that was shifting eastward toward the sea (Figure 2d,g). To the north and northwest of the city, a broad low-pressure system prevailed. This east-high–west-low pressure gradient between Central China (e.g., Henan and Hubei) and the FWP established a strong, sustained southeasterly wind. The southeasterly flow extended upward to 850 hPa (Figure 2e,h), corresponding to the mid-upper boundary layer on the southwestern side of an intensified high-pressure system—a system that evolved from the powerful high observed on 21 September. This synoptic configuration dynamically reinforced the low-level southeasterly current, forming a deep, persistent southeasterly transport corridor stretching from the Central China Plain directly into the Guanzhong Basin. At 700 hPa (Figure 2f,i), the circulation remained dominated by the same high-pressure system, which favored the long-range transport of pollutants along the corridor while inhibiting their vertical dispersion into the free troposphere.

As shown in Figure S1, wind fields during the pre-outbreak phase (23–25 September) were notably weaker than those on polluted days. A near-reversal in surface wind direction occurred between 23 and 24 September, driven by a fundamental shift in the pressure pattern over XY resulting from the movement of high-pressure systems. Wind speeds during this period were significantly lower than those on 27 September; such unfavorable dispersion conditions favored the accumulation of pollutants in the region. However, these stagnant conditions were alleviated on 25 September, when the southeastward shift in a high-pressure system to the northeast and the southward movement of a low-pressure system from the north established a steady southeasterly flow with significantly stronger wind speeds. Pollutant accumulation peaked on that day as the transport corridor began to take shape, signaling the imminent onset of regionally transported pollution.

In contrast, synoptic conditions and source regions on typical clean days were fundamentally different. On 21 September (Figure 2a–c), XY was mainly influenced by northeasterly flow ahead of a continental high-pressure system. Air masses originated from the relatively clean Inner Mongolia region, effectively blocking the inflow of pollutants from eastern and southern sources. On 30 September (Figure 2j–l), the area exhibited typical post-cold-frontal conditions, with a strong cold high-pressure system dominating northern China. Under this synoptic regime, robust northwesterly winds prevailed over the FWP, characterized by high horizontal wind speeds and excellent atmospheric dispersive capacity, which rapidly removed both local and upwind residual pollutants, resulting in excellent air quality.

### 3.2. O_3_ Transport Flux Calculation

The discussion above confirms that horizontal and vertical transport induced by large-scale circulation evolution serves as the key dynamical mechanism driving local O_3_ pollution in XY. In this section, we draw on vertical O_3_ profiles from O_3_ lidar and vertical dimension wind observations from a wind profile radar to trace O_3_ and wind fields and to calculate the horizontal and vertical transport fluxes of O_3_.

**Figure 3:**
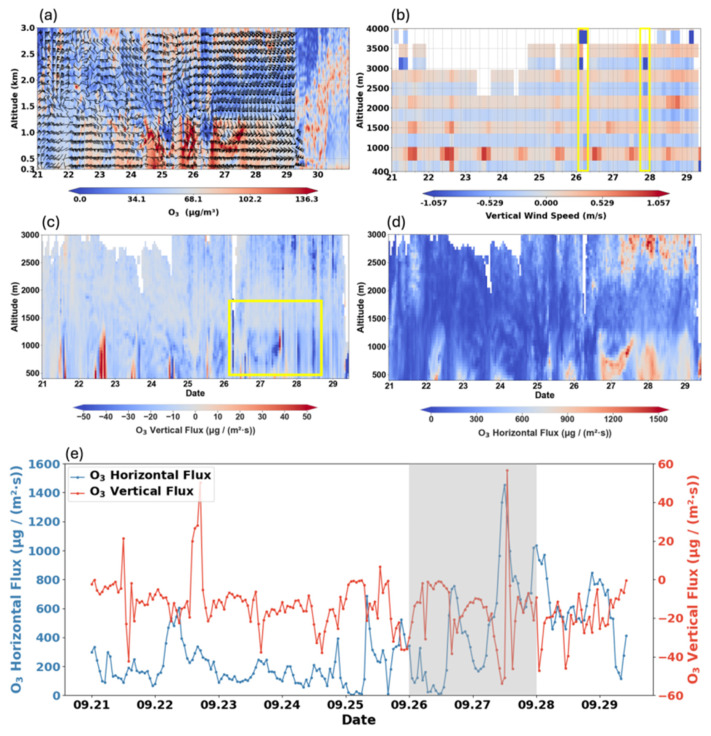
(**a**) O_3_ lidar profiles (µg/m^3^) and horizontal winds (m/s); (**b**) vertical wind speed (m/s); (**c**) vertical O_3_ flux (µg/(m^2^·s)); (**d**) horizontal O_3_ flux (µg/(m^2^·s)); and (**e**) time series of horizontal and vertical O_3_ flux at 1000 m (µg/(m^2^·s)). The gray shaded area denotes the heavy pollution episode.

Figure 3 quantifies the physical transport process driven by this synoptic system, presenting vertical profiles of O_3_ concentration and wind fields, three-dimensional fluxes, and their temporal evolution. Coordinated measurements from O_3_ lidar and wind-profile radar (Figure 3a) delineate the internal structure of the transport channel during 26–27 September, a layer of elevated O_3_ maintained stability between 300 and 1500 m, with concentrations exceeding 100 µg/m^3^. This pattern stands in stark contrast to the low-background conditions seen on clean days and the weaker pre-episode signals. Wind vectors within this layer aligned closely with the southeasterly flow at 850 hPa shown in Figure 2, confirming that the elevated O_3_ originated directly from directional upstream transport.

The evolution of O_3_ fluxes derived from O_3_ lidar and wind-profile radar observation further quantifies the transport intensity within this channel. As shown in Figure 3d, strong horizontal fluxes were concentrated within the southeasterly wind layer between 500 and 1500 m, with peak values exceeding 1200 µg/(m^2^·s). The time series at 1000 m reveals an explosive increase in horizontal flux during the episode, reaching an instantaneous maximum of 1580 µg/(m^2^·s) on the afternoon of 27 September (Figure 3e). This value was markedly higher than the background levels during clean periods (<400 µg/(m^2^·s)), indicating that substantial amounts of O_3_ were continuously advected into the region, which became the dominant driver for the rising O_3_ concentrations.

Vertical O_3_ flux confirms the subsequent downward transport of O_3_. During daytime on polluted days, strong negative fluxes peaked above –151.5 µg/(m^2^·s) within the mid-to-upper boundary layer (yellow box in Figure 3c), reflecting efficient downward mixing of O_3_ that further intensified surface pollution in XY. In contrast, strong positive fluxes prevailed near the surface on 23–24 September, with peaks exceeding 69.6 µg/(m^2^·s), which helped suppress the accumulation of surface O_3_ during those non-polluted days. The observed subsidence was dynamically driven by divergence and sinking motions within the high-pressure system (Figure 2).

Overall, the combined use of O_3_ lidar and wind-profiler radar provides direct evidence for reconstructing the spatiotemporal evolution of O_3_. These observations substantiate that O_3_ transport from upwind source regions, mediated by both horizontal advection and vertical components, contributed decisively to the elevated O_3_ pollution levels in XY.

### 3.3. Variational Characteristics of O_3_ Precursors Sensitivity

Previous studies have indicated that regional transport can not only elevate the O_3_ level but also carry O_3_ precursors, thereby altering OPS [44]. To examine the evolution of OPS during the pollution episode, we analyzed the spatiotemporal distributions of HCHO and NO_2_ using GEMS satellite remote sensing data and assessed the dynamic changes in OPS.

**Figure 4:**
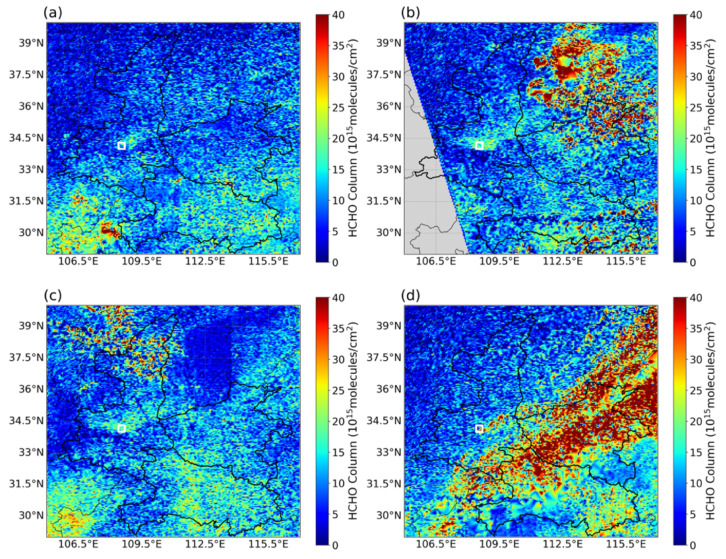
Spatial distribution of tropospheric HCHO column densities (10^15^ molecules/cm^2^) over XY and surrounding regions derived from GEMS satellite observations. (**a**–**d**) correspond to 21, 26, 27 and 30 September 2024, respectively. The white square indicates the location of XY.

**Figure 5:**
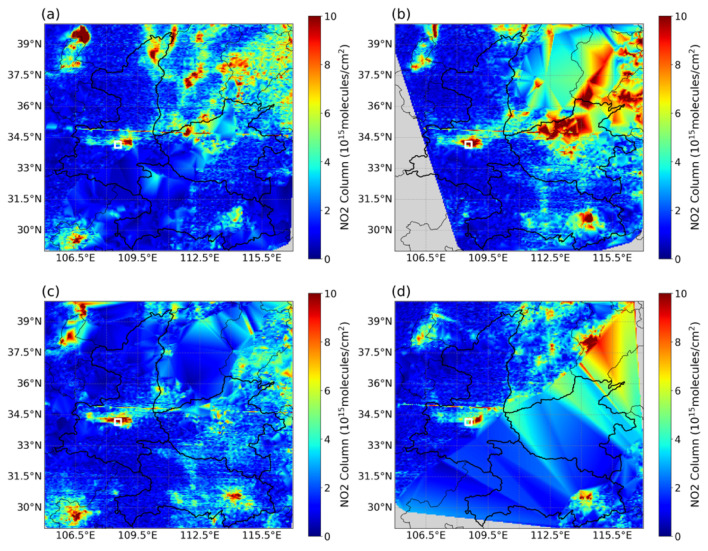
Spatial distribution of tropospheric NO_2_ column densities (10^15^ molecules/cm^2^) over XY and surrounding regions derived from GEMS satellite observations. (**a**–**d**) correspond to 21, 26, 27 and 30 September 2024, respectively. The white square indicates the location of XY.

Figure 4 and Figure 5 depict the spatial accumulation of precursors over XY and its surrounding regions during the pollution episode. On 26–27 September, satellite observations revealed pronounced hotspots of HCHO (Figure 4b,c) and NO_2_ (Figure 5b,c). Together with the daily evolution shown in Figures S2 and S3, we noticed that the periphery of the HCHO hotspots migrated toward XY, resulting in an elevation of HCHO vertical column densities from 5–10 × 10^15^ to 10–15 × 10^15^ (molecules/cm^2^). Focusing on the local XY area (marked by the white box), the satellite-derived column densities of HCHO and NO_2_ on the polluted day (26 September) increased by 157% compared with those on the clean day (21 September) (Figure 4a and Figure 5a).

**Figure 6:**
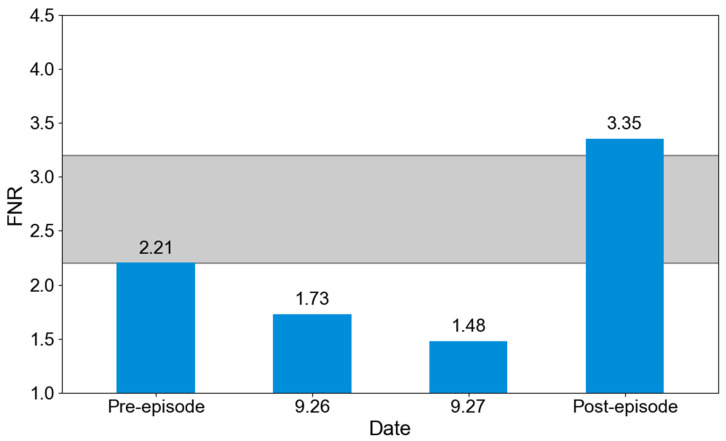
Formaldehyde-to-nitrogen dioxide ratio (FNR) in the XY region during different phases. The OPSs are classified as VOC-limited (FNR < 2.2), transitional (2.2 ≤ FNR ≤ 3.2; gray color), and NO_X_-limited (FNR > 3.2). The periods shown are before (21–25 September) and after (28–30 September) the O_3_ episode.

The ratio of HCHO to NO_2_ column concentrations (FNR = HCHO/NO_2_) was used to diagnose shifts in the O_3_ formation sensitivity regime (Figure 6). We employed satellite HCHO to calculate the FNR indicator for analysis primarily due to its complete spatial coverage of HCHO and NO_2_ across the Fenwei Plain, which is essential for our study scale. This approach simultaneously leverages a well-established and widely used methodological framework [45,46]. Under the influence of air masses transported from HB and HN, the FNR in XY changed notably: during the early phase of the pollution episode, the FNR was 2.21 (within the transitional regime), whereas in the later phase it increased to 3.35, indicating a shift to a NO_X_-limited regime where O_3_ production is primarily controlled by NO_X_ availability. On the most severely polluted days (26 and 27 September), FNR dropped to 1.73 and 1.48, well below the threshold of 2.2, identifying a VOC-limited regime. This pattern suggests that strong regional transport not only contributes directly to O_3_, but also significantly alters the local VOC/NO_X_ balance due to the differing chemical characteristics of incoming air masses [47,48]. Although satellite observations clarify the macro-scale transition of O_3_ sensitivity driven by regional transport, they cannot directly quantify the contributions from individual upwind provinces or identify the specific emission sectors responsible for precursor enhancements. Therefore, further integration with numerical models is required to perform refined source attribution—both geographically and by sector.

### 3.4. O_3_ Source Appointment Analysis

**Figure 7:**
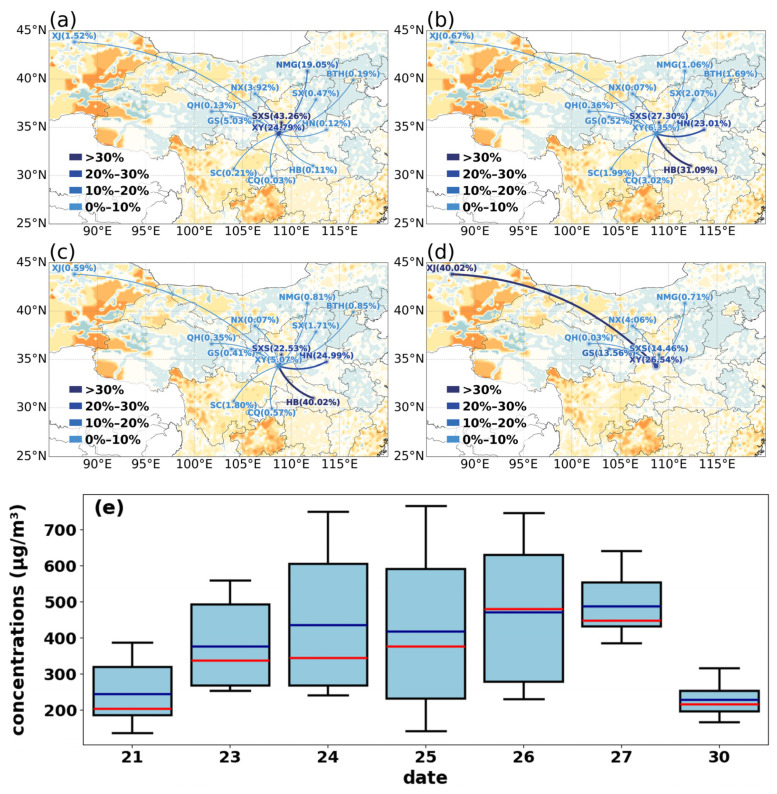
The regional contribution to daily O_3_ in XY for (**a**) 21, (**b**) 26, (**c**) 27, and (**d**) 30 September. (**e**) atmospheric oxidative capacity (µg/m^3^) from 21 and 23–30 September. The red and blue lines indicate the median and mean value, respectively.

Building on the observational evidence highlighting the significant role of regional transport in O_3_ enhancement, this section further quantifies the contributions from different source regions and characterizes the emission properties of the transported air masses. By using the OSAM technology, we quantify the potential sources of O_3_ in XY. During clean periods, the regional atmospheric environment was dominated by local emissions and northwesterly background flows. On 21 September (Figure 7a), O_3_ originated mainly from within Shaanxi Province (SXS, 43.26%) and local XY (24.79%), while on 30 September (Figure 7d), O_3_ levels were primarily attributable to long-range transport from the northwest, specifically Xinjiang (XJ, 29.71%) and Inner Mongolia (NMG), alongside local contributions. The HYSPLIT simulation results presented in Figure S4 effectively validate the accuracy of the OSAM model.

As shown in Figure 7, the background shading represents the VOC/NO_X_ emission ratio obtained from the Multi-resolution Emission Inventory for China (MEIC) which provides data at a monthly temporal resolution. We specifically utilized the emission inventory for September.

During the typical polluted days (26–27 September), the source structure underwent a fundamental shift, with local contributions sharply declining and cross-regional transport becoming the dominant driver (Figure 7b,c). The contribution rate from local XY dropped below 10%, while the contribution from southeasterly transport rose substantially. Notably, on 27 September, the contribution rates from HB and HN provinces reached 40.02% and 24.99%, respectively, together accounting for over 65% of the total O_3_ concentration. Furthermore, the VOC/NO_X_ emission ratios in the HN and HB regions were lower than those observed in SXS, with the ratios in SXS mostly ranging from 2.0 to 2.5, whereas those in HB and HN were predominantly within the range of 0.5 to 2.0. (Figure 7). This indicates that the southeasterly winds transported NO_X_-enriched air from HB and HN into XY, while the scavenging effects of strong winds mitigated the local accumulation of pollutants by promoting their regional export [49] and simultaneously removed local air masses with higher VOC proportions, thereby altering the local OPS and enhancing the local AOC. Regarding O_3_ control, upstream regions such as HN and HB contributed over 50% to this episode, suggesting that individual local emission reductions are insufficient to curb such transport-oriented pollution. To improve air quality in XY and the Guanzhong Plain during the O_3_ season, it is essential to establish a cross-regional joint prevention and control mechanism covering the Guanzhong–HN–HB corridor and to prioritize the management of industrial zones with high VOC/NO_X_ ratios along the transport channel.

The regional transport process not only directly imports high concentrations of O_3_, but also substantially enhances local AOC. Total oxidant (O_X_ = O_3_ + NO_X_) is widely adopted as a more robust indicator of photochemical aging and oxidative potential than O_3_ alone, as it effectively accounts for the titration effect of NO [50]. As shown in Figure 4a, atmospheric oxidation in XY intensified markedly during the pollution episode. On clean days, median and mean O_X_ concentrations remained relatively low (~200 µg/m^3^), reflecting weak background oxidative capacity. In contrast, under the influence of southeasterly transport on 26–27 September, median O_X_ surged to between 450 and 480 µg/m^3^, with occasional peaks exceeding 600 µg/m^3^, indicating a highly oxidative state. This elevated AOC accelerated the oxidation of gaseous precursors (e.g., SO_2_ and NO_X_) into secondary inorganic aerosols (SIA) and promoted the aging of VOCs and the formation of secondary organic aerosols (SOA) [51,52], which aligns with the rise in PM_2.5_ concentrations observed on polluted days (Figure 1c).

**Figure 8:**
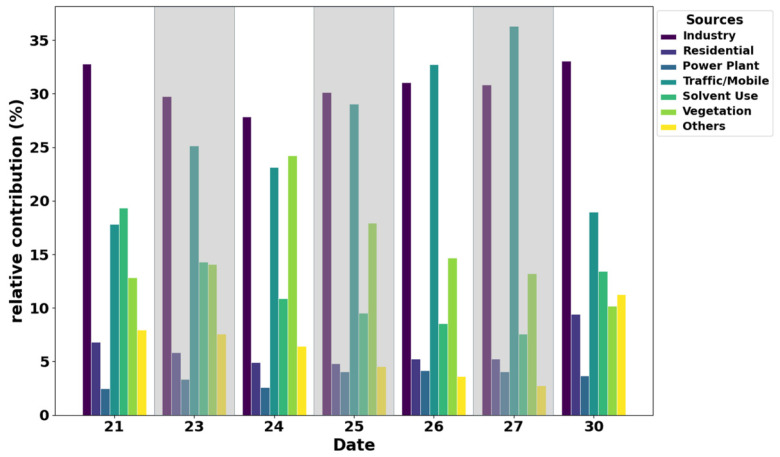
Daily contributions to O_3_ concentration in different sources simulated by NAQPMS.

To characterize the emission sources of the regionally transported air masses, O_3_ were classified into seven source categories using the NAQPMS. As shown in Figure 8, the O_3_ source profile in XY exhibited pronounced temporal variations throughout the sampling period. The industrial sector showed notable persistence, with its contribution consistently ranging between 30% and 35%. This high and stable share indicates that industrial emissions form the background O_3_ load in the region, largely due to the continuous operation of industrial processes, whose emission intensity is less influenced by diurnal meteorological variations compared to mobile or biogenic sources.

Notably, beginning on 25 September, the contribution from the transportation sector increased markedly, surpassing that of industrial sources for the first time on 26 September and peaking at 36% on 27 September, when it became the dominant O_3_ source. This rise was driven by the regional transport of vehicle exhaust and its secondary products from upwind urban agglomerations, such as the Wuhan and Central Plains metropolitan areas, into XY. The increase in the transportation sector’s contribution correlated strongly with the observed rise in particulate nitrate (NO_3_^−^) and ammonia (NH_3_) (Figure 1b), as motor vehicles are one of the major sources of urban NO_X_ [53], a key precursor of secondary nitrate, and gasoline vehicles with three-way catalysts are an important urban source of NH_3_. These findings provide a scientific basis for formulating regional joint prevention and control strategies, as well as coordinated O_3_ mitigation measures in XY.

## 4. Conclusions

Since the full implementation of the Blue Sky Protection Campaign in 2018, the FWP has been designated as one of the new key regions for national air pollution control. By integrating multi-source Observations technologies, including O_3_ lidar, wind-profiler radar, and GEMS satellite remote sensing, with chemical transport modeling, this study elucidates the driving mechanisms through which cross-regional transport influences local O_3_ pollution outbreak.

During the pollution episode, a strong southeasterly flow established a well-defined O_3_ transport corridor over XY. Quantitative vertical observations revealed a distinct high-O_3_ layer (concentrations > 130 µg/m^3^) within the mid- to upper boundary layer (300–1500 m), confirming that advection from Central China was the primary driver of pollution buildup. More importantly, measured vertical fluxes showed pronounced subsidence (peaking below −100 µg/(m^2^·s)), delineating the dynamic process through which elevated O_3_ and its precursors were effectively entrained and transported downward to the surface by turbulent mixing and sinking motions. It is worth noting that, in addition to O_3_ and VOCs, the long-range transport of PM_2.5_ is also an important feature of regional pollution. Unlike HCHO, which has a relatively short atmospheric lifetime, PM_2.5_ can remain in the atmosphere for several days [54]. Transported aerosols may indirectly affect local O_3_ production through aerosol-radiation interactions or heterogeneous chemical processes [55,56]. Additionally, enhanced atmospheric oxidants resulting from O_3_ pollution promote the formation of secondary PM_2.5_.

Regional transport critically modulated local AOC and OPS. Integrated satellite and ground-based observations revealed a distinct chemical transition induced by the southeasterly inflow. Owing to the differing precursor composition of the transported air masses, the local FNR value decreased from 1.9 before the outbreak to 1.48 during the episode, shifting the OPS further into the VOC-limited regime and thereby increasing the sensitivity of local O_3_ formation to VOC emissions. Concurrently, transport substantially enhanced the AOC, with peak O_X_ levels exceeding 600 µg/m^3^. This elevated AOC accelerated the secondary conversion of SO_2_ and NO_X_ into secondary inorganic aerosols (SIA), resulting in pronounced synergistic pollution of both O_3_ and PM_2.5_.

OSAT results indicate that cross-regional transport dominated the O_3_ episode, with local contributions falling below 10% on the most polluted days. The major external sources were upwind HB of 40.02% and HN of 24.99%, which together accounted for over 65% of the O_3_. In terms of sectoral contributions, while industrial emissions constituted the regional background (~30%), the transported air masses carried a strong mobile-source signature, with transportation surpassing industry as the leading contributor on the peak pollution day. In light of the shift toward a VOC-limited regime and the elevated role of transportation emissions, conventional control measures that focus only on local industrial sources or single-pollutant (e.g., NO_X_) reductions are likely to be insufficient. We therefore recommend establishing a large-scale regional joint prevention and control framework covering the Guanzhong–HN–HB corridor. During autumn and summer pollution episodes, a differentiated strategy emphasizing VOC reduction and coordinated management of mobile sources along the transport pathway should be prioritized. For example, increasing efforts to promote the use of electric vehicles and public transportation in surrounding areas.

In summary, this study employs multi-source three-dimensional observational data to quantify the contribution of regional transport to O_3_ pollution and underscores the complexity and urgency of managing transboundary air pollution. It reveals that conventional administrative-boundary-based governance models exhibit considerable limitations in addressing O_3_ episodes characterized by elevated transport pathways. The integrated observation and source-apportionment framework established here provides a robust scientific foundation for implementing precise cross-provincial joint prevention and control, offering a relevant case for advancing regional air quality management strategies in China.

## Data Availability

The original contributions presented in this study are included in the article/Supplementary Materials. Further inquiries can be directed to the corresponding author.

## Supplementary Materials

The following supporting information can be downloaded at: https://www.mdpi.com/article/10.3390/toxics14030189/s1, Figure S1: Synoptic circulation patterns of sea-level pressure, 10 m wind speed, and geopotential height at surface, 850 hPa, and 700 hPa for clean days on 23 September (a–c); 24 (d–f) and 25 (g–i). The asterisk denotes the central location of XY; Figure S2: Spatial distribution of tropospheric HCHO column densities (10^15^ molecules/cm^2^) over XY and surrounding regions derived from GEMS satellite observations. Panels (a–c) correspond to 23, 24 and 25 September 2024, respectively. The white square indicates the location of XY; Figure S3: Spatial distribution of tropospheric NO_2_ column densities (10^15^ molecules/cm^2^) over XY and surrounding regions derived from GEMS satellite observations. Panels (a–c) correspond to 23, 24 and 25 September 2024, respectively. The white square indicates the location of XY; Figure S4: Results of HYSPLIT simulated air mass backward trajectories for XY on (a) 21, (b) 26, (c) 27, and (d) 30 September.

## Abbreviations

The following abbreviations are used in this manuscript:

O_3_	Ozone
PM_2.5_	Particulate matter with <2.5 µm aero-dynamic diameters (PM_2.5_)
FWP	Fenwei Plain
HYSPLIT	Single-Particle Lagrangian Integrated Trajectory
VOCs	Volatile organic compounds
MDA8 O_3_	Daily maximum 8 h average O_3_
OSAM	O_3_ source apportionment method
NAQPMS	Nested Air Quality Prediction Modeling System
SOA	Secondary organic aerosols
XY	Xianyang province
XJ	Xinjiang province
SAX	Shaanxi province
HN	Hunan province
HB	Hubei province
QH	Qinghai province
SX	Shanxi province
SC	Sichuan province
NX	Ningxia province
GS	Gansu province
CQ	Chongqing
BTH	Beijing–Tianjin–Hebei Region
FRN	Formaldehyde to nitrogen dioxide ratio
