# Supplementary material for: Impacts of Cross-Regional Transport on Ozone Pollution in the Fen-Wei Plain: Insights from Multi-Source Observations and Model Simulation
Source: Toxics. 2026 Feb 24;14(3):189. doi: 10.3390/toxics14030189 (PMC13030664; doi:10.3390/toxics14030189)
Supplement: Supplementary file 1 [file toxics-14-00189-s001.zip › toxics-4114860-supplementary.pdf]

toxics

MDPI

Supplement of

Impacts of Cross-Regional Transport on Ozone pollution in the Fen-Wei Plain: Insights from multi-source Observations and Model simulation

Yufei Han [1], Danni Xu [1,2], Anjie Yin [1], Chang Liu [1], Yuheng Chen [1], and Kaihui Zhao [1*]

[1] Yunnan Key Laboratory of Meteorological Disasters and Climate Resources in the Greater Mekong Subregion, Yunnan University, Kunming, 650091, China
[2] Information school, Yunnan University of finance and economics, Kunming, 650221, China
* Correspondence: khzhao@ynu.edu.cn

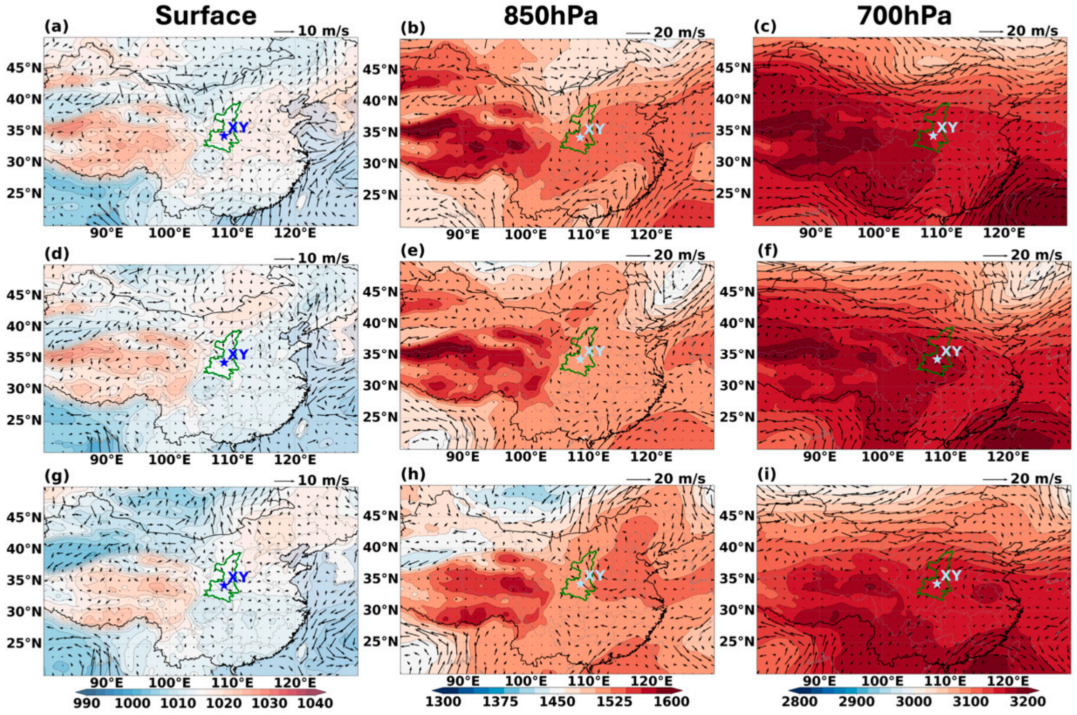

Figure S1. Synoptic circulation patterns of sea-level pressure, 10 m wind speed, and geopotential height at surface, 850 hPa, and 700 hPa for clean days on September 23 (**a, b, c**); 24 (**d, e, f**) and 25 (**g, h, i**). The asterisk denotes the central location of XY.

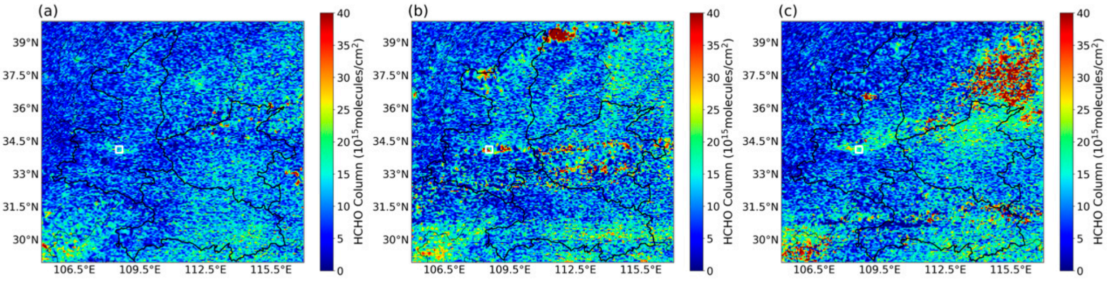

Figure S2. Spatial distribution of tropospheric HCHO column densities (10^{15} molecules/cm^2) over XY and surrounding regions derived from GEMS satellite observations. Panels (**a**)–(**c**) correspond to September 23, 24 and 25, 2024, respectively. The white square indicates the location of XY.

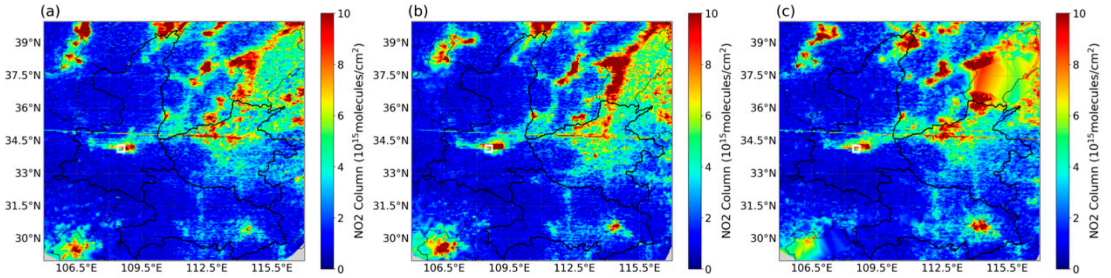

Figure S3. Spatial distribution of tropospheric NO_2 column densities (10^{15} molecules/cm^2) over XY and surrounding regions derived from GEMS satellite observations. Panels (**a**)–(**c**) correspond to September 23, 24 and 25, 2024, respectively. The white square indicates the location of XY.

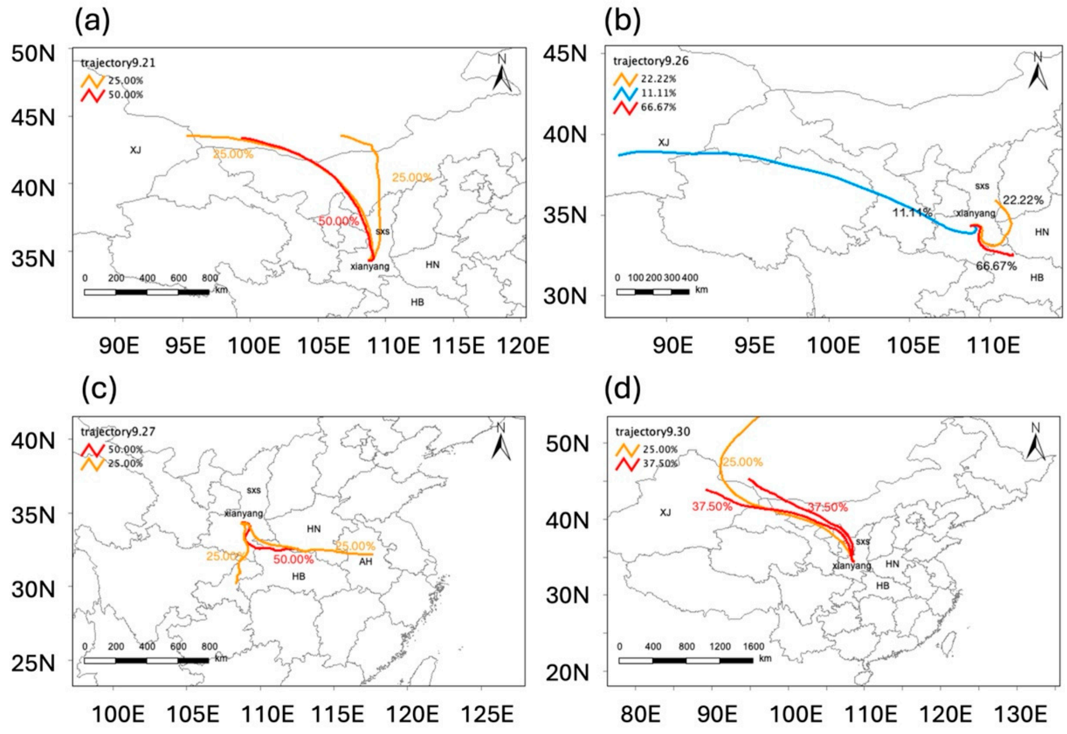

Figure S4. Results of HYSPLIT simulated air mass backward trajectories for XY on September (a) 21, (b) 26, (c) 27, and (d) 30.
